# Combining metabolomics and transcriptomics to characterize tanshinone biosynthesis in *Salvia miltiorrhiza*

**DOI:** 10.1186/1471-2164-15-73

**Published:** 2014-01-28

**Authors:** Wei Gao, Hai-Xi Sun, Hongbin Xiao, Guanghong Cui, Matthew L Hillwig, Alana Jackson, Xiao Wang, Ye Shen, Nan Zhao, Liangxiao Zhang, Xiu-Jie Wang, Reuben J Peters, Luqi Huang

**Affiliations:** 1 National Resource Center for Chinese Materia Medica, Academy of Chinese Medical Sciences, Beijing 100700, China; 2 School of Traditional Chinese Medicine, Capital Medical University, Beijing 100069, China; 3 State Key Laboratory of Plant Genomics, Institute of Genetics and Developmental Biology, Chinese Academy of Sciences, Beijing 100101, China; 4 Division of Biotechnology, Dalian Institute of Chemical Physics, Chinese Academy of Sciences, Dalian 116023, China; 5 Department of Biochemistry, Biophysics & Molecular Biology, Iowa State University, Ames, Iowa 50011, USA

## Abstract

**Background:**

Plant natural products have been co-opted for millennia by humans for various uses such as flavor, fragrances, and medicines. These compounds often are only produced in relatively low amounts and are difficult to chemically synthesize, limiting access. While elucidation of the underlying biosynthetic processes might help alleviate these issues (e.g., via metabolic engineering), investigation of this is hindered by the low levels of relevant gene expression and expansion of the corresponding enzymatic gene families. However, the often-inducible nature of such metabolic processes enables selection of those genes whose expression pattern indicates a role in production of the targeted natural product.

**Results:**

Here, we combine metabolomics and transcriptomics to investigate the inducible biosynthesis of the bioactive diterpenoid tanshinones from the Chinese medicinal herb, *Salvia miltiorrhiza* (Danshen). Untargeted metabolomics investigation of elicited hairy root cultures indicated that tanshinone production was a dominant component of the metabolic response, increasing at later time points. A transcriptomic approach was applied to not only define a comprehensive transcriptome (comprised of 20,972 non-redundant genes), but also its response to induction, revealing 6,358 genes that exhibited differential expression, with significant enrichment for up-regulation of genes involved in stress, stimulus and immune response processes. Consistent with our metabolomics analysis, there appears to be a slower but more sustained increased in transcript levels of known genes from diterpenoid and, more specifically, tanshinone biosynthesis. Among the co-regulated genes were 70 transcription factors and 8 cytochromes P450, providing targets for future investigation.

**Conclusions:**

Our results indicate a biphasic response of Danshen terpenoid metabolism to elicitation, with early induction of sesqui- and tri- terpenoid biosynthesis, followed by later and more sustained production of the diterpenoid tanshinones. Our data provides a firm foundation for further elucidation of tanshinone and other inducible natural product metabolism in Danshen.

## Background

Plant extracts have been used as flavor, fragrances, and medicines for millennia. More recently, it has been possible to trace these desirable properties to specific natural products. However, these often are not found in large amounts in the native producing plants. Moreover, in many cases, these turn out to be complex compounds not readily accessible by synthetic means. Thus, their use is limited by availability. In addition, the full potential of promising natural products is restrained by these same factors, which prevents not only direct investigation, but also semi-synthetic optimization of their desirable properties (e.g., pharmacological). These issues potentially could be relieved by elucidation of the relevant biosynthesis – e.g., to enable metabolic engineering to provide access to the targeted natural product or variants thereof either in the native host or recombinant systems. However, our ability to carry out such investigations has been hindered by the limited information generally available for the plant(s) of interest.

The genus *Salvia* contains almost 1,000 identified species, many of which are well known for their aromatic properties and/or pharmological uses, which are attributable to a wealth of specialized metabolites, mainly terpenoids and phenylpropanoids. Many of these species are traditionally used as medicinal herbs. For example, *Salvia miltiorrhiza*, also known as “Danshen”, has recorded medical usage dating back to nearly two thousand years ago. Danshen is an important traditional Chinese medicine, the rhizome of which has been used extensively for the treatment of coronary heart diseases, particularly angina pectoris and myocardial infarction [1]. The tanshinones are abietane-type norditerpenoid quinones that make up the bioactive lipophilic pigments from the intensely red rhizome of *S. miltiorrhiza* and exhibit a variety of pharmaceutical effects, including antibacterial, anti-inflammatory, and broad antitumor activities [1,2]. This has been attributed to their inhibition of the hypoxia-inducible factor 1 [3], negative regulation of the PI3K signaling pathway [4], and/or inhibition of the Aurora A kinase [5]. Due to their important medicinal activity, chemical syntheses of tanshinones and their analogs have attracted great attention since the early 1960s [6], but these are still limited by low yields [2]. On the other hand, hairy root cultures of *S. miltiorrhiza* make tanshinones [7], where their production can be induced [8-11], providing a model system for investigation of tanshinone biosynthesis [12].

As terpenoids, the tanshinones originate from more general isoprenoid metabolism. In plants, the isoprenoid precursors isopentenyl diphosphate (IPP) and dimethylallyl diphosphate (DMAPP) are derived from two distinct pathways, the mevalonate (MVA) pathway operating in the cytosol, and the 2-C-methyl-D-erythritol 4-phosphate (MEP) pathway occurring in plastids [9,13,14]. While the biosynthesis of diterpenoids is initiated in plastids, cross-talk between the MVA and MEP pathways has been shown [14], and tanshinone production has been shown to be reduced by the MVA pathway inhibitor mevinolin [9], as well as stimulated by overexpression of the key MVA pathway enzyme 3-hydroxy-3-methylglutaryl CoA reductase (HMGR) [15]. Nevertheless, the tanshinones are mainly derived from the MEP pathway [9].

Due to its medical importance, tanshinone biosynthesis has been heavily investigated. This includes some expressed sequence tag (EST) studies of Danshen hairy root cultures induction [16,17]. These studies led to the identification of some enzymes from the MVA pathway [15], and, more critically, enzymes specific to tanshinone biosynthesis. In particular, the relevant cyclases, copalyl diphosphate synthase (SmCPS) and kaurene synthase-like (SmKSL), which together catalyze the formation of miltiradiene from the general diterpenoid precursor geranylgeranyl diphosphate (GGPP), with miltiradiene representing a plausible precursor to the tanshinones [18]. Recently, application of a modular pathway engineering (MOPE) strategy has led to significant production levels of miltiradiene in the yeast *Saccharomyces cerevisiae*, providing a platform on which further investigations can be carried out [19].

Despite these advances, further elucidation of tanshinone biosynthesis has been retarded by the limited sequence information available for *S. miltiorrhiza*. The previous EST studies identified only a few thousand genes, representing < 20% of the expected transcriptome, and there is no genome sequence yet available [2]. Given our interest in obtaining a deeper understanding of tanshinone metabolism, we undertook a combined metabolomic and transcriptomic investigation of the elicitation process in *S. miltiorrhiza* hairy root cultures that leads to the production of tanshinones.

## Results

### Metabolomic analysis of induced *S. miltiorrhiza* hairy root cultures

**Figure 1:**
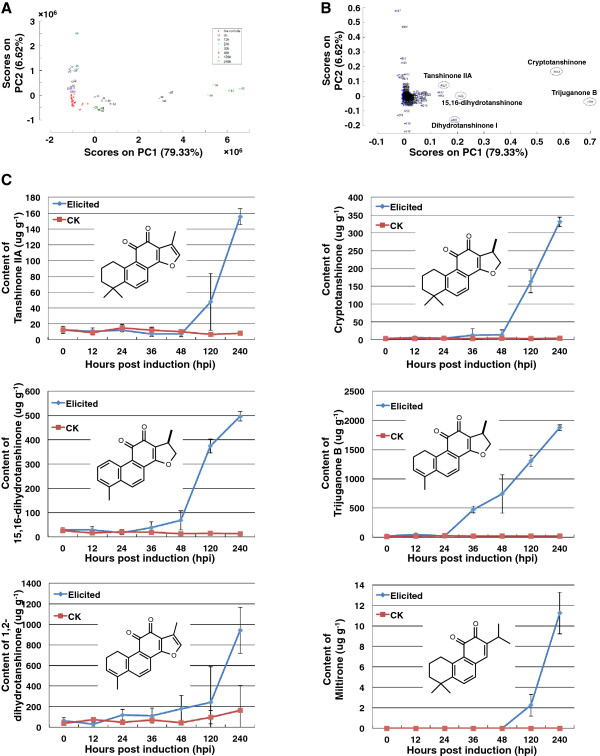
**Tanshinone production is a major metabolic response of *S. miltiorrhiza *hairy root cultures to induction. (A and B)** Plot of the first two components, accounting for almost 86% of the total variability, from principle component analysis (PCA) of the detected *S. miltiorrhiza* metabolites. **(A)** Shown are compounds whose peak accumulation occurs at the indicated time, demonstrating that the first principal component mainly reflects metabolites produced at later time points, whereas the second principal component represents changes at earlier time points. **(B)** The five compounds most representative to the first principal component are tanshinones, namely tanshinone IIA, cryptotanshinone, 15,16-dihydrotanshinone, trijuganone B, and dihydrotanshinone I. **(C)** Plots demonstrating the increasing accumulation of miltirone and the five compounds identified in panel B over time (error bars represent standard error of the mean, SEM). Also shown are the corresponding structures.

We developed *S. miltiorrhiza* hairy root cultures by infecting sterile plantlets (collected from ShangLuo Shanxi province, PRC) with a Ri T-DNA bearing *Agrobacterium rhizogenes* (ATCC15834). Such hairy roots are homogeneous and known to accumulate tanshinones, providing an ideal model system for studying tanshinone biosynthesis [7]. Using the previously reported combined biotic (yeast extract) and abiotic (Ag^+^) induction method [9], the expected strong accumulation of tanshinones in these hairy root cultures was observed (see Additional file 1: Figure S1). We conducted a large-scale non-targeted metabolite analysis using ultra-performance liquid chromatography coupled with diode array detection and quadrupole time-of-flight mass spectrometry (UPLC-DAD-QTOF-MS) to identify functional components secreted by *S. miltiorrhiza* following elicitation. A total of 3,862 unfiltered peaks were detected from the obtained data using the MZmine LC-MS toolbox [20]. Principal components analysis (PCA) [21] on mean centered data identified two major principle components that could clearly separate the elicited and control samples (Figure 1A). The first principal component mainly reflected changes at 36 or more hrs post induction (hpi), which accounted for over 79% of the total variability, whereas the second principle component mainly reflected earlier changes (Figure 1A). Hierarchical clustering analysis demonstrated that the expression of many tanshinone related compounds exhibited dramatic increases at 120 hpi and 240 hpi (see Additional file 2: Figure S2 and Additional file 3: Table S1). Moreover, five of these metabolites were identified from the PCA loading plot to have the most significant contribution to the first principal component, namely tanshinone IIA, cryptotanshinone, 15,16-dihydrotanshinone, trijuganone B, and dihydrotanshinone I (Figure 1B). Examination of ultra-performance liquid chromatography (UPLC) results showed that the content of all these metabolites increased significantly at the later examined time points, especially at 120 hpi and 240 hpi (Figure 1C and see Additional file 4: Table S2).

### Transcriptomic analysis of induced *S. miltiorrhiza* hairy root cultures

**Figure 2:**
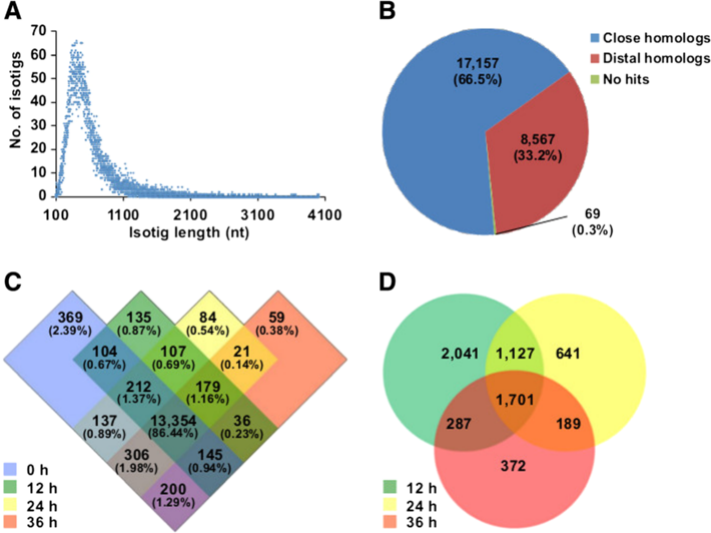
**mRNA profiling of *S. miltiorrhiza *hairy roots by high-throughput sequencing. (A)** Length distribution of the 25,793 identified isotigs. **(B)** Summary of automated annotation. Close homologs refers to isotigs with BLASTX hits that exhibit ≥ 30% identity over ≥ 30% of the sequence, distal homologs refer to isotigs whose BLASTX hits do not met this criteria, while no hits refer to isotigs without any BLASTX hits. **(C)** Venn diagram showing the number (and the corresponding percentage of the total) of genes expressed at the various time points. **(D)** Venn diagram showing the number of differentially expressed (DE) genes found at the various time points.

As the genome sequence of *S. miltiorrhiza* is not yet available, and we were most interested in transcribed genes in any case, we carried out transcriptomic analysis of the induced *S. miltiorrhiza* hairy root cultures. Previous investigations of rice diterpenoid biosynthesis had demonstrated that transcriptional responses precede phytochemical accumulation [22-24]. Accordingly, we focused on earlier time points following elicitation of the *S. miltiorrhiza* hairy root cultures (≤ 36 hpi). We first used the Roche 454 sequencing technology to generate a reference transcriptome from a pooled cDNA library (i.e., from all the time points). This yielded 1,061,065 reads, totaling 193,983,972 bases, which were assembled into a total of 25,793 non-redundant isotigs with lengths largely ranging from 100 to 1,100 nt (Figure 2A). Putative gene functions were assigned to these isotigs by comparing them to the NCBI nr database (non-redundant protein database) using the BLASTX program. Among the 25,793 isotigs, 17,157 (66.5%) had homologs with ≥ 30% sequence identity in the nr database (close homologs), 8,567 (33.2%) only had BLAST hits with sequence identities lower than this threshold (distal homologs), and the remaining 69 (0.3%) had no hit in the nr database, suggesting they might be undiscovered genes or *S. miltiorrhiza* and/or *Salvia* specific genes (Figure 2B). By merging isotigs with overlapping sequences and closely related, putative alleles and/or homoeologs, a final total of 20,972 non-redundant genes were obtained (see Additional file 5: Table S3). Given that the total length of these genes was 11,850,070 bases, our 454 sequencing data represents just over 16-fold coverage of this reference transcriptome.

The transcriptional response of *S. miltiorrhiza* hairy root cultures to induction was determined by an RNA-seq approach, using cDNA libraries generated from non-induced (0 hpi) and 12 hpi, 24 hpi and 36 hpi cultures using an Illumina GAII sequencer, providing 36 nt long single-end reads. A total of 6,882,388, 6,300,372, 5,731,519 and 5,690,024 reads were obtained from the 0 hpi, 12 hpi, 24 hpi and 36 hpi libraries, respectively. From these, about 72-75% of the reads from each time point were perfect matches to the isotigs from our reference transcriptome (see Additional file 6: Table S4), covering 87.1% of the total nr isotigs (see Additional file 7: Table S5). We next determined the expression levels of each gene by calculating Reads Per Kilobase exon model per Million mapped reads (RPKM) values. Using an threshold of RPKM expression values ≥ 2, we found that about 68% of the genes were expressed in each cDNA library (see Additional file 8: Table S6). Among these, ~86.4% were expressed at all time points, and over 50% of these genes had log-2 transformed RPKM values greater than 4 (Figure 2C and see Additional file 9: Figure S3). Using a 2-fold difference in RPKM and a Fisher’s exact test p-value of less than 0.05 as cutoffs, 5,156, 3,658 and 2,549 genes were identified as differentially expressed (DE) at the 12 hpi, 24 hpi and 36 hpi time points as compared to their level in the uninduced (0 hpi) control, respectively, representing a total of 6,358 DE genes (Figure 2D and see Additional file 10: Table S7).

### Functional analysis of DE genes

**Figure 3:**
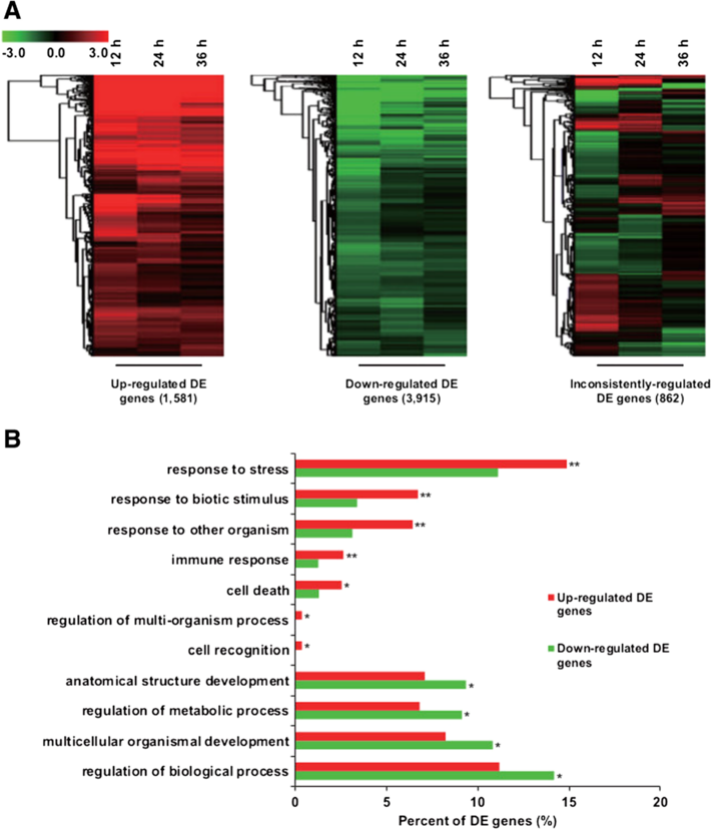
**Functional analysis of differentially expressed (DE) genes. (A)** Hierarchically clustered heat map for the expression profile of DE genes (reflecting log-2 transformed expression fold changes relative to the control) that are either only up-regulated (left) or down-regulated (middle), as well as the remaining DE genes, whose expression changes in an inconsistently manner (right). **(B)** Plot of gene ontology (GO) terms (from the third level of biological process category) for the DE genes falling into either the consistently up- (red) or down- (green) regulated categories. Enrichment was measured by comparing the number of genes from each DE category to the total number of genes for that GO term and using Fisher’s exact test, significance is indicated for resulting p-values below 0.01 or between 0.01 and 0.05 by double or single asterisks, respectively.

To investigate the functions of the 6,358 DE genes, we grouped them into three categories according to their relative expression profiles following induction, namely those that were only up-regulated, only down-regulated, and those with inconsistent changes in their expression level, (Figure 3A and see Additional file 11: Table S8). Gene Ontology (GO) analysis revealed that GO terms related to stress, stimulus, and immune response processes were significantly enriched among the up-regulated DE genes (p-value < 0.05, Fisher’s exact test) (Figure 3B). By contrast, genes related to development and metabolic processes were mostly down-regulated after induction (Figure 3B).

**Figure 4:**
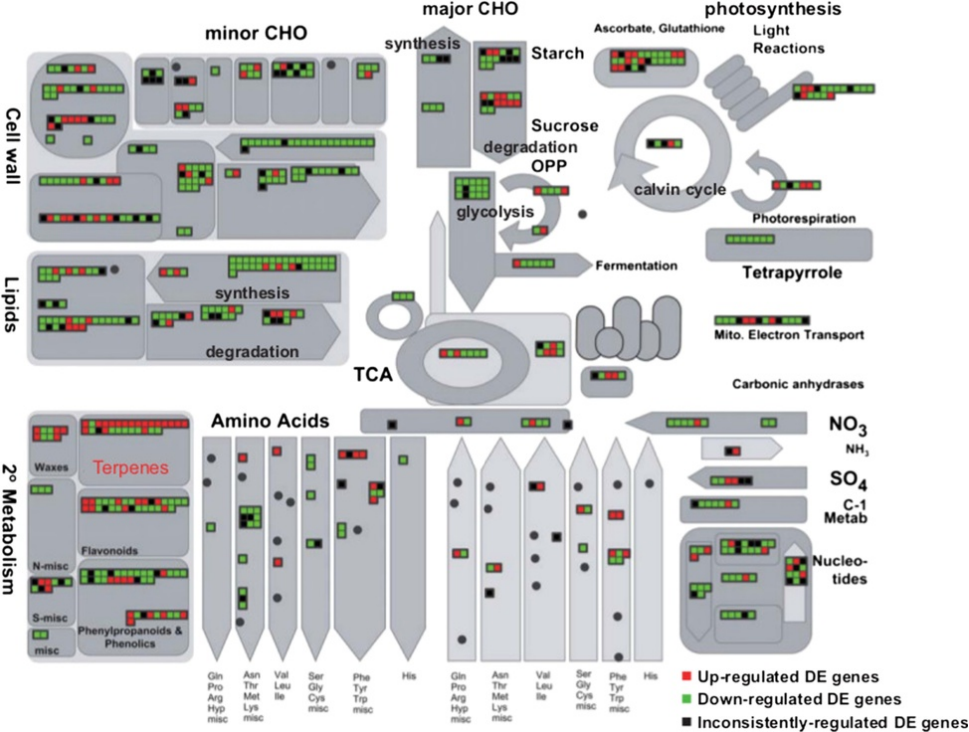
**Overview of induced transcriptional changes for the enzymatic genes from various metabolic pathways.** Each square represents a DE gene.

To analyze the relationship of DE genes with metabolic processes, we used the MapMan tool [25] to visualize the distribution of DE genes on known metabolic pathways (Figure 4). Consistent with the GO analysis results (Figure 3B), the expression of genes related to central metabolic pathways, such as photosynthesis, lipid and nucleotide metabolism, were repressed after induction (Figure 4). By contrast, many genes involved in terpenoid metabolism were up-regulated.

### Analysis of terpenoid metabolism

**Figure 5:**
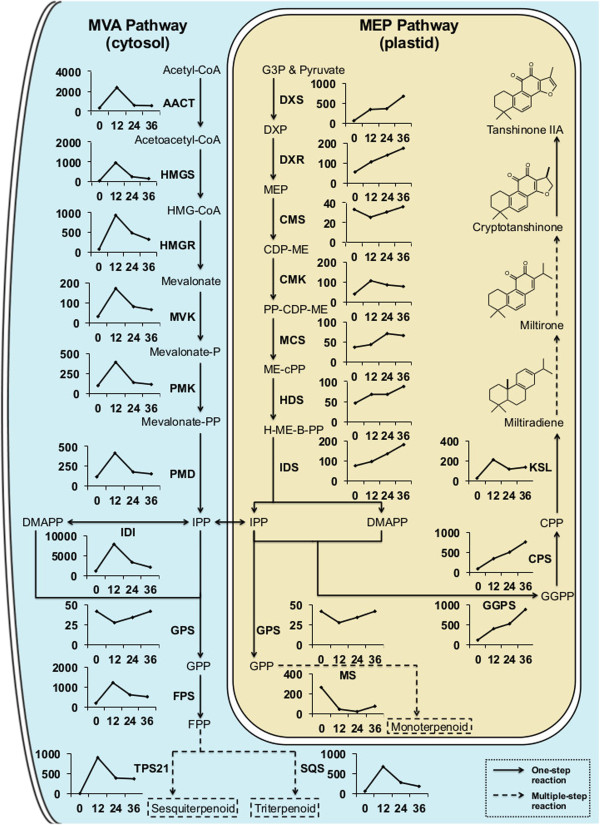
**Expression profile for the genes involved in terpenoid biosynthesis.** Enzyme abbreviations are presented in bold capital letters, next to the corresponding catalyzed reaction (arrow). The relevant expression pattern is also shown as a line plot, where the X-axis represents hours post induction (hpi) and the Y-axis represents the RPKM expression value. Solid arrows represent one-step reactions, and dashed arrows represent multiple-step reactions.

The production of tanshinones in *S. miltiorrhiza* involves the formation of isoprenoid precursors, as well as diterpenoid biosynthesis more specifically. Accordingly, we first inspected the expression of genes from the upstream isoprenoid precursor biosynthetic pathways, namely the cytosolic MVA pathway and the plastidial MEP pathway, in our RNA-seq data. All MVA pathway related genes exhibited a significant increase in expression levels at 12 hpi, but this was followed by a significant drop at 24 hpi, and a return to expression levels only slightly higher than the control (0 hpi) at 36 hpi (Figure 5), representing a rapid but transient response to elicitation. On the other hand, most genes in the MEP pathway exhibited more gradual, yet significant, increased expression levels, most of which are still increasing at the last 36 hpi time point, exhibiting the expected correlation to tanshinone production (Figure 5). The expression profile of these genes was confirmed by qRT-PCR analysis (see Additional file 12: Figure S4).

Terpenoids are sub-divided on the basis of the number of constituent five-carbon isoprenyl units, with the ten-carbon monoterpenoids generally derived from geranyl diphosphate (GPP), the fifteen-carbon sesquiterpenoids from farnesyl diphosphate (FPP), the twenty-carbon diterpenoids from GGPP, and the thirty-carbon triterpenoids from squalene. In plants, the biosynthesis of sesquiterpenoids and triterpenoids is initiated in the cytoplasm, whereas that of the monoterpenoids and diterpenoids is initiated in plastids. The expression pattern of the FPP synthase (FPS) and squalene synthase (SQS) in our RNA-seq data resembles that of the upstream MVA pathway, as does that of a putative sesquiterpene cyclase (TPS21) also identified among the DE genes (Figure 5). Intriguingly, expression of the GPP synthase (GPS) is not significantly changed during induction, and that of a putative monoterpene cyclase (MS) found among the DE genes is actually significantly down-regulated. By contrast, expression of the GGPP synthase (GGPS) is steadily and very significantly increased throughout the transcriptomic time course, as is that of SmCPS, with significantly increased levels of SmKSL observed as well. All of these are more specifically involved in tanshinone biosynthesis (Figure 5), and their expression pattern is consistent with the observed later and sustained production of these diterpenoid natural products (Figure 1C and see Additional file 2: Figure S2).

### Identification of co-regulated cytochrome P450 genes

**Figure 6:**
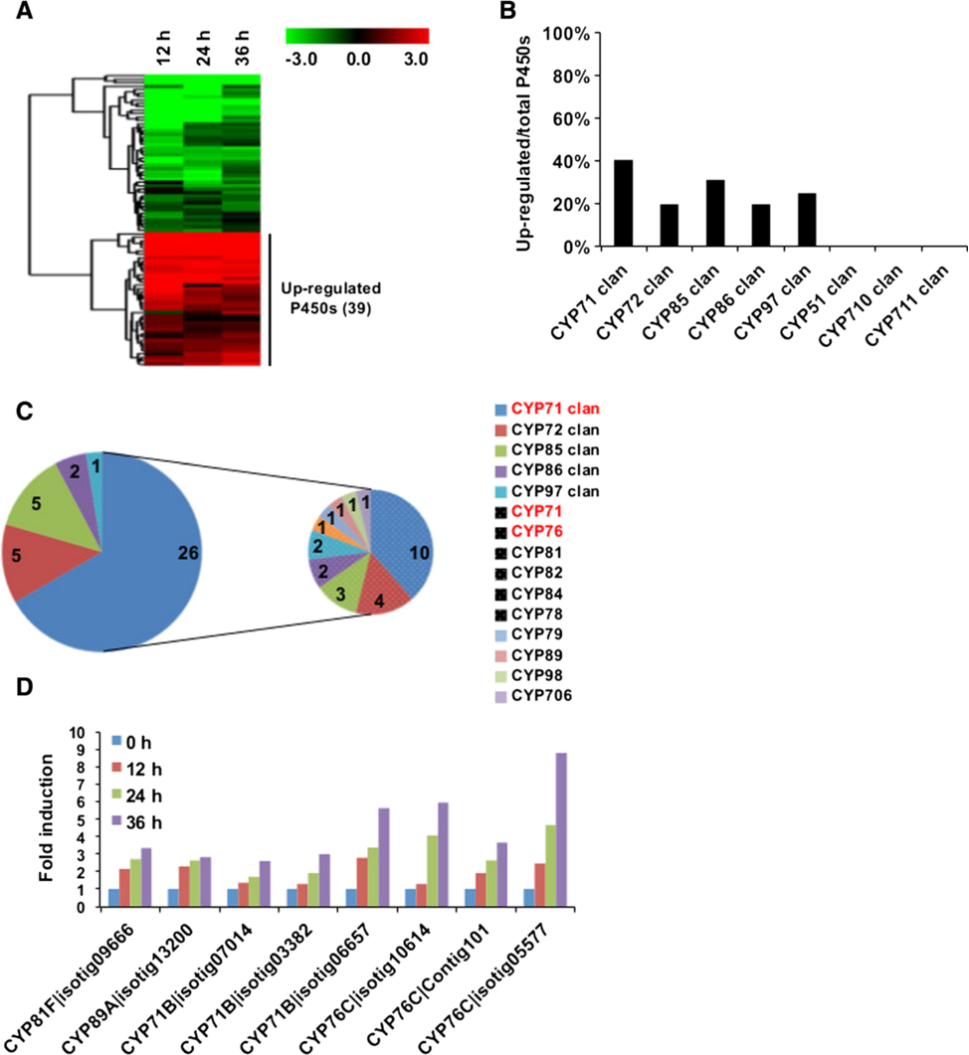
**Candidate cytochrome P450 genes for tanshinone biosynthesis. (A)** Hierarchically clustered heat map for the 85 DE cytochrome P450 (CYP) genes reflecting log-2 transformed expression fold changes relative to the control). **(B)** Proportion of up-regulated genes from each CYP clan. **(C)** Distribution of the 39 up-regulated CYPs in clans, as well as the family distribution of the 26 members from the CYP71 clan. **(D)** Histogram plot of the fold induction at each time point for each of the 8 CYP genes exhibiting continuously increasing expression levels following induction.

Cytochrome P450s (CYPs) monooxygenases play a key role in terpenoid biosynthesis, with such activity almost invariably required for further transformation of olefinic intermediates such as miltiradiene, but as yet their roles in tanshinone biosynthesis are not well characterized. The CYPs are divided into related families (designated by number) and more loosely related clans [26], and our RNA-seq data revealed 125 expressed CYP genes, covering 8 clans and 31 families (Table S9). From these, 85 were among the DE genes, with the transcript levels of 39 found to be increased at all time points following induction (Figure 6A). The CYP71 clan was the most up-regulated group of CYPs, with about 40% of the genes from this clan exhibiting increased transcript levels after induction (Figure 6B). Within this clan, genes from the CYP71 and CYP76 families accounted for more than half of those up-regulated. This seems to be due, at least in part, to the relatively large size of these families (Figure 6C and see Additional file 13: Table S9), which is consistent with the family distribution of CYPs found in other plants species [27]. We hypothesize that transcription of the CYPs involved in tanshinone biosynthesis will be co-regulated with that of the already identified enzymatic genes – i.e., by continuously increasing expression after induction (e.g., SmCPS, see Figure 5), which highlights eight CYP genes. All of these belonged to the CYP71 clan (Figure 6D), members of which are generally involved in plant natural products biosynthesis [26].

### Expression profiling of transcription factor family genes

Given the transcriptional regulation of plant natural products metabolism described above, identification of the relevant transcription factors (TFs) for the observed induction of tanshinone biosynthesis also is of clear interest. By comparison with the TFs from *Arabidopsis thaliana* in PlantTFDB, we identified a total of 1,162 expressed TFs from our transcriptome [28]. Among these, 412 TFs belonging to 45 families were among the DE genes (see Additional file 14: Table S10). In general, the 12 hpi and 24 hpi samples shared more TF genes with common expression changes than the 24 hpi and 36 hpi samples (see Additional file 15: Figure S5A). The differentially expressed TFs were grouped into three categories by the k-means clustering method. This revealed that there were 68 TFs whose expression was consistently down-regulated, 70 TFs that were consistently up-regulated, and 274 genes with inconsistent changes in expression (see Additional file 15: Figure S5B-D). Many of the down-regulated TFs were related to plant development regulation, such as the basic/helix-loop-helix (bHLH) and homeodomain-leucine zipper (HD-ZIP) family genes. By contrast, most of the up-related TFs were related to stress response, as exemplified by the heat shock factors and some WRKY family genes (see Additional file 15: Figure S5B-D).

**Figure 7:**
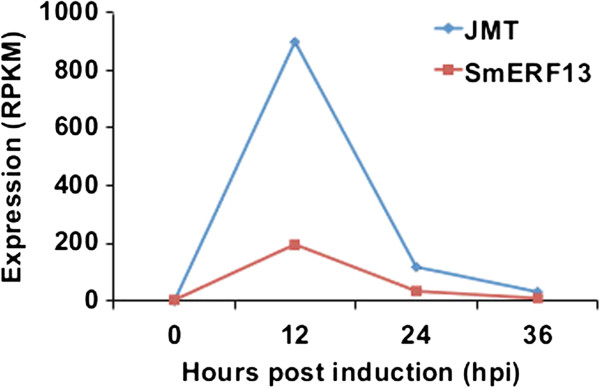
**Correlation of JMT and SmERF13 with tanshinone biosynthesisc Co-expression of JMT and SmERF13 in response to induction.** The X-axis represents hours post induction (hpi) and the Y-axis represents the RPKM expression value.

It has previously been shown that application of the plant defense signaling molecule methyl jasmonate (MeJA) increases tanshinone production in hairy root cultures [29]. However, the relevance of this to the elicitation method utilized here was uncertain. Transcription of the jasmonic acid carboxyl methyltransferase (JMT), which directly produces MeJA [30], was strongly increased during induction. In particular, while JMT transcripts were not detected in the control (0 hpi) culture, its transcript reached an expression level of over 900 RPKM at 12 hpi (Figure 7A), suggesting that elicitation may enhance endogenous MeJA biosynthesis via induction of JMT. Moreover, it has been shown that the downstream response to MeJA is mediated, at least in part, by TF from the ERF family, whose own transcription is inducible by MeJA – e.g., AtERF13 in *Arabidopsis thaliana*[31], and ORCA3 in *Catharanthus roseus*, which is involved in activating the terpenoid indole alkaloid (TIA) biosynthesis pathway [32]. Notably, examination of our differentially expressed TFs revealed an AtERF13 and ORCA3 homolog, SmERF13, whose expression is significantly increased during induction (Figures 7 and see Additional file 15: Figure S5).

## Discussion

*S. miltiorrhiza*, also known as Danshen, is an important traditional Chinese medicinal herb whose intensely red rhizome has been used to treat heart diseases for millennia, and extracts of which are currently in clinical trials [33]. The characteristic pigmentation is imparted by the tanshinones, a group of abietane-type norditerpenoids with various pharmaceutical activities [1]. Despite extensive efforts tanshinones remain only poorly accessible via synthesis and their supply is, thus, limited by the natural variation inherent in agricultural production. While this restriction might be alleviated by knowledge of the underlying biosynthetic pathway (e.g., to enable metabolic engineering in Danshen and/or microbial production hosts), little is known about tanshinone biosynthesis. Progress has been retarded, in part, by the limited sequence information available for *S. miltiorrhiza*, as well as some means to associate particular genes with tanshinone biosynthesis. Here we have taken advantage of the inducible nature of tanshinone production in hairy root cultures to take a combined metabolomic and transcriptomic approach towards alleviating both issues.

We first carried out metabolomic analysis of hairy root culture exudates harvested either before (0 hpi control) or at various times following induction. PCA demonstrated that the first component, accounting for almost 80% of the observed differences, was correlated with metabolites appearing at later (≥ 36 hpi) time points (Figure 1). Notably, the metabolites most correlated with the first component are tanshinones, whose levels are strongly elevated at 120 hpi and 240 hpi, although increases can be seen as early as 36 hpi (Figure 1C).

To provide more complete sequence information, we turned to deep sequencing – i.e., transcriptomics. With the expectation that transcription precedes production, we concentrated our sequencing efforts on earlier time points in the induction process (≤ 36 hpi). A reference transcriptome consisting of 20,972 non-redundant genes was obtained from a pooled cDNA library using the longer read 454 technology, while an RNA-seq approach using Illumina technology was used to analyze the change in this transcriptome during elicitation (Figure 2). Mapping the 6,358 differentially expressed genes (Figure 3) onto general metabolism revealed a global pattern consisting of down-regulation of central metabolism, along with up-regulation of terpenoid biosynthesis (Figure 4).

Closer examination of the expression of genes from terpenoid metabolism demonstrated a striking biphasic response. First, an early, but transient, up-regulation of genes from the cytosolic MVA isoprenoid precursor pathway, along with genes involved in the sesqui- and tri- terpenoid metabolism also found in the cytosol. This is followed by a more gradual, but sustained, up-regulation of genes from the plastidial MEP isoprenoid precursor pathway. Such transcriptional up-regulation of the isoprenoid precursor pathways, which is likely to increase flux to terpenoid natural products [34], has been previously observed – e.g., in rice, although there only the MEP pathway was reported to be up-regulated by elicitation [35]. Thus, the bi-phasic response of the MVA and MEP pathways observed here may be specific to elicitation of *S. miltiorrhiza* hairy root cultures.

In any case, the gradual sustained induction of the plastidial MEP pathway seems to be relevant to tanshinone biosynthesis, as genes from the monoterpenoid metabolism also found in the plastids are down-regulated, while the few genes already identified in tanshinone biosynthesis (i.e., SmCPS and SmKSL) clearly exhibit the same sustained increase in transcript levels (Figure 5). On this basis, we have further identified eight CYP whose similar transcriptional pattern suggests a potential role in tanshinone biosynthesis (Figure 6). Finally, we also mined our RNA-seq data to identify transcriptional factors potentially involved in eliciting tanshinone biosynthesis, as well as further suggest a role for the defense signaling molecule methyl jasmonate in the induction process (Figure 7).

## Conclusions

The combined metabolomics and transcriptomic approach utilized here has provided some insight into the observed inducible nature of tanshinone production in hairy root cultures of *S. miltiorrhiza*. For example, highlighting a potential role for the induced, methyl jasmonate-responsive transcription factor SmERF13 in regulating such elicitation. Perhaps more critically, our combined metabolomics and transcriptomics data has revealed a distinct expression pattern correlated with tanshinone production, which provides a firm foundation for further investigation of the biosynthesis of these medically important natural products.

## Methods

### Hairy root culture development and induction system

Hairy root cultures were obtained by infecting sterile *S. miltiorrhiza* plantlets with a Ri T-DNA Agrobacterium rhizogenes (ATCC15834). Induction was started 18 days after inoculating 2 g fresh weight of hairy roots in 250 ml Erlenmeyer flasks by the application of a biotic-abiotic combination of the carbohydrate fraction of yeast extract (100 μg ml^-1^) with Ag^+^ (30 μM) as previously described [9]. Hairy roots were harvested at 0 h, 12 h, 24 h, 36 h, 48 h, 120 h, and 240 h post induction, from three individual cultures at each time point, which were divided into two parts, one stored at −80°C for transcriptome profiling, the other stored at −20°C for metabolite analysis.

### Extraction and sample preparation

Total RNA was extracted from pooled hairy roots (i.e., all three cultures) at 0 h, 12 h, 24 h, 36 h and 48 h post induction using the Trizol method (Invitrogen, Carlsbad, CA, USA). In addition, a modified version of a previously described protocol was employed for preferential extraction of tanshinones from lyophilized hairy roots (n = 3) at 0 h, 12 h, 24 h, 36 h, 48 h, 120 h, and 240 h post induction [36]. Briefly, after ultrasound lysis in 20 ml of methanol/chloroform (7:3 vol/vol) for 60 min, the extracts were centrifuged at 2500 r min^-1^ for 2 min and the supernatant was removed and dried down. The residue was subsequently dissolved in 2 ml of methanol. This solution was filtered through a 0.22 μm micropore membrane prior to use.

### Ultra-performance liquid chromatography coupled with diode array detection and quadrupole time-of-flight mass spectrometry (UPLC-DAD-QTOF-MS) analysis

Metabolite analyses were carried out using an Agilent 1290 Infinity HPLC system equipped with a binary pump, a diode array detector (DAD), an autosamper, and a column compartment. After testing, a Poroshell 120 SB-C18 column (3.0 × 150 mm, 2.7 um, Agilent) was chosen for optimal separation. The mobile phase was formed from solvent A [0.5% (vol/vol) formic acid aqueous solution containing 10% acetonitrile] and B [0.5% (vol/vol) formic acid methanol solution containing 10% acetonitrile]. The column was eluted using a gradient of 10% to 100% solvent B (vol/vol) over 10 min, then 100% B for the next 5 min, at a flow rate of 0.8 mL/min at 65°C. The HPLC chromatogram was monitored at 280 nm. MS detection was carried out with an Agilent 6520 Q-TOF mass spectrometer with an Atmospheric Pressure Chemical Ionization (APCI) interface. The ion source in the positive ion mode was operated at 3500 V cap and 4 μA corona current. Drying gas and vaporizer temperature were set at 350°C and 325°C, respectively. The nebulizer pressure was 50 psi, with drying gas at 5 L/min. For full-scan MS analysis, the spectra were recorded in the range of 100–1000 m/z. Each of the three biological replicates (independent cultures from each time point) was analyzed in triplicate chromatographic runs (i.e., technical replicates).

### Metabolic profile analysis

The metabolic profiles of the *S. miltiorrhiza* hairy roots were analyzed using a previously described LC/MS data protocol [37]. Briefly, after transforming the raw Agilent data into MZ-mine format, automatic integration and peak alignment were conducted for subsequent explorative data analysis. Principal component analysis (PCA) was employed to investigate the difference between elicited groups and non-elicited groups, as well as the time series changes in detected metabolites. Hierarchical clustering was used to examine the relationship among these metabolites over the sampled time series (shown as a heat map in Additional file 2: Figure S2). Data analysis was performed using MZ-mine LC/MS tools and MATLAB.

### Roche 454 and Illumina GAII sequencing and data analysis

To generate a reference transcriptome, total RNA isolated from hairy roots collected at 0 h, 12 h, 24 h, 36 h and 48 h post induction were pooled for cDNA synthesis. Roche 454 FLX sequencing was carried out on cDNAs isolated from the 500–700 nt size range. After removing the adapter sequences, cleaned sequence reads were assembled using the CAP3 software [38]. Individual isotigs were annotated by searching the NCBI non-redundant protein sequence (nr) database using the BLASTX software with default parameters. Isotig functions were assigned based on the annotation associated with the top hit that satisfied the following criteria: (i) ≥ 30% sequence identity; (ii) ≥ 30% alignment coverage of either the query or subject sequences; and (iii) with BLAST e-values < 1e^-5^. After merging isotigs with overlapping sequences, a total of 20,972 non-redundant genes were obtained. The sequences of these genes (in FASTA format) can be found in Table S3 (see Additional file 5). For Illumina GAII sequencing, total RNA isolated from the hairy roots collected at 0 h, 12 h, 24 h and 36 h post induction were individually used for 3′ fragment cDNA synthesis. With each sample, Illumina GAII sequencing was carried out with cDNAs isolated in the size range of 250–450 nt. After removing the sample identifying sequence tags, the resulting Illumina sequencing reads were mapped to the reference transcriptome by the SOAP software (Release 2.20, 08-13-2009) [39]. The expression levels of isotigs at each of the examined time point was evaluated by the RPKM value of the Illumina sequencing reads according to the following equation: $\mathrm{RPKM}=\frac{\mathrm{total}\;\mathrm{exon}\;\mathrm{reads}}{\mathrm{mappable}\;\mathrm{reads}\;\left(\mathrm{million}\right)\times \mathrm{exon}\;\mathrm{length}\;\left(\mathrm{Kb}\right)}$. GO annotations of genes were assigned using InterProScan [40]. MapMan software (version 3.5.1) was used to visualize the relative expression level of genes with assigned metabolic function. KEGG pathway analysis was carried out using KAAS [41]. Hierarchical clustered heat maps were produced with MEV (version 4.6.2) [42]. Multiple sequence alignments were generated using ClustalW2 [43].

Expression of homologous genes of the same gene family was calculated according to the abundance of reads uniquely mapped to each gene. The gene from each gene family with the highest expression value was chosen to represent the expression of that gene family in the figures reported here.

### Annotation of transcription factor families

Similar to the procedure of NCBI nr annotation, the assembled isotigs were searched against the TAIR9 protein database using the BLASTX software with default parameters. The sequences of the top hits were compared with the sequences of annotated *Arabidopsis thaliana* transcription factors from PlantTFDB [28]. Expression profiles of transcription factors were clustered using log-2 transformed expression fold changes at each time point as compared to the control by k-means clustering method with MEV (version 4.6.2) [42].

### Quantitative real-time PCR

For each qRT-PCR reaction 200 ng of total RNA was used as the template and the reaction carried out using the PrimeScriptTM 1st Strand cDNA Synthesis Kit (Takara, Tokyo, Japan) and Power SYBR Master Mix (Applied Biosystems, Foster City, California, USA) with gene-specific primer pairs (see Additional file 16: Table S11). To estimate the relative mRNA level, a series of diluted reference cDNA samples were used as control templates. The relative amounts of the target genes were evaluated by the relative expression index of mRNA using the 2(−△△C(T)) Method [44]: F = 10(△CT,T/AT) − (△CT,R/AR), where T represents the target gene, R refers to β-Actin, and △CT is the difference in the threshold cycle value (CT).

### Availability of supporting data

Roche 454 sequencing data has been deposited in the National Center for Biotechnology Information (NCBI) Sequence Read Archive (SRA) under accession SRX317052. Illumina GAII sequencing data has been deposited in the NCBI SRA under accession SRX317054.

## Abbreviations

AACT: Acetyl-CoA C-acetyltransferase; Acetoacetyl-CoA: Acetoacetyl coenzyme A; Acetyl-CoA: Acetyl coenzyme A; bHLH: basic/helix-loop-helix; CDP-ME: 4-(Cytidine 5′-diphospho)-2-C-methyl-D-erythritol; CMK: 4-(Cytidine 5′-diphospho)-2-C-methyl-D-erythritol kinase; CMS: 4-(Cytidine 5′-diphospho)-2-C-methyl-D-erythritol synthase; CPS: Copalyl diphosphate synthase; CYPs: Cytochrome P450s; DE genes: Differentially expressed genes; DMAPP: Dimethylallyl pyrophosphate; DXP: 1-Deoxy-D-xylulose 5-phosphate; DXR: 1-deoxy-D-xylulose-5-phosphate reductoisomerase; DXS: 1-deoxy-D-xylulose-5-phosphate synthase; EST: Expressed sequence tag; FPP: Farnesyl diphosphate; FPS: FPP synthase; G3P: Glyceraldehyde 3-phosphate; GGPP: Geranylgeranyl diphosphate; GGPS: GGPP synthase; GO: Gene Ontology; GPP: Geranyl diphosphate; GPS: GPP synthase; HDS: 4-hydroxy-3-methylbut-2-enyl diphosphate synthase; HD-ZIP: Homeodomain-leucine zipper; H-ME-B-PP: 1-Hydroxy-2-methyl-2-butenyl 4-diphosphate; HMG-CoA: 3-hydroxy-3-methylglutaryl CoA; HMGR: 3-hydroxy-3-methylglutaryl CoA reductase; HMGS: Hydroxymethylglutaryl-CoA synthase; hpi: Hours post induction; IDI: Isopentenyl-diphosphate delta-isomerase; IDS: Isoprenyl diphosphate synthase; IPP: Isopentenyl pyrophosphate; JMT: Jasmonic acid carboxyl methyltransferase; KSL: Kaurene synthase-like; MCS: 2-C-methyl-D-erythritol 2,4-cyclodiphosphate synthase; ME-cPP: 2-C-Methyl-D-erythritol 2,4-cyclodiphosphate; MeJA: Methyl jasmonate; MEP: 2-C-methyl-D-erythritol 4-phosphate; Mevalonate-P: Mevalonate-5-phosphate; Mevalonate-PP: Mevalonate-5-Diphosphomevalonate; MOPE: Modular pathway engineering; MS: Monoterpene cyclase; MVA: Mevalonate; MVK: Mevalonate kinase; PMD: Diphosphomevalonate decarboxylase; PMK: Phosphomevalonate kinase; PP-CDP-ME: 2-Phospho-4-(cytidine 5′-diphospho)-2-C-methyl-D-erythritol; RPKM: Reads Per Kilobase exon model per Million mapped reads; S. miltiorrhiza: *Salvia miltiorrhiza*; SQS: Squalene synthase; TFs: Transcription factors; TIA: Terpenoid indole alkaloid; TPS21: Terpene synthase 21; UPLC: Ultra-performance liquid chromatography; UPLC-DAD-QTOF-MS: Ultra-performance liquid chromatography coupled with diode array detection and quadrupole time-of-flight mass spectrometry.

## Competing interests

The authors declare that they have no competing interests.

## Authors’ contributions

The project was designed and supervised by XJW, RJP and LH; WG, GC and YS prepared the RNA samples for transcriptome sequencing; WG, HX, XW, NZ and LZ carried out the metabolome analysis; WG carried out the qPCR analysis; HXS, AJ and MLH carried out bioinformatics analyses; WG, HXS, MLH, XJW, RJP and LH all participated in writing the paper. All authors read and approved the final manuscript.

## Supplementary Material

Additional file 1: Figure S1 Induced accumulation of tanshinones in elicited *S. miltiorrhiza* hairy root cultures. The observed red color is due to the accumulation of tanshinones. Thus, the notably deeper red coloration after induction relative to the control group indicates a significant increase in tanshinone content (which has been verified by LC/MS analysis – e.g. see Figures 1 and S2). Click here for file

Additional file 2: Figure S2 Metabolomic analysis of elicited *S. miltiorrhiza* hairy roots by ultra-performance liquid chromatography coupled with diode array detection and quadrupole time-of-flight mass spectrometry (UPLC-DAD-QTOF-MS). A final set of 179 peaks was obtained from the metabolite profile after peak filtering analysis. The depicted heat map represents hierarchical clustering of the log-2 transformed levels for all 179 compounds. Included among these are many tanshinones related compounds (shown in red), namely miltirone, cryptotanshinone, dihydrotanshinone, trijuganone A, trijuganone B, tanshinone IIA, tanshinone IIB, tanshinone VI, ferruginol, hydrotanshinone IIA, 1,2-dihydrocrytotanshinone, etc. Click here for file

Additional file 3: Table S1 LC/MS chromatographic data at each time point for the 179 peaks whose abundances are shown in Additional file 2: Figure S2 (response is averaged from triplicate runs for each of the three biological replicates/samples for each time point). Click here for file

Additional file 4: Table S2 Tabulated data for the tanshinone abundance levels depicted in Figure 1C. Click here for file

Additional file 5: Table S3 The sequences of the 20,972 non-redundant genes identified here (FASTA format). Click here for file

Additional file 6: Table S4 Summary of sequence reads with matches to the reference *S. miltiorrhiza* transcriptome. Click here for file

Additional file 7: Table S5 Summary of *S. miltiorrhiza* isotigs with matched sequence reads. Click here for file

Additional file 8: Table S6 Summary of genes detected in each sample (i.e., time points post induction). Click here for file

Additional file 9: Figure S3 Plot of gene expression abundance. The X-axis represents log-2 transformed RPKM values and the Y-axis represents the accumulating proportion of genes observed with at least that level of expression. Click here for file

Additional file 10: Table S7 List of up- and down-regulated DE genes at the various time points post induction. Click here for file

Additional file 11: Table S8 Tabulated data of RPKM values and associated p-value for significance of any observed change, assignment of observed changes and DE gene category, sequence length, and annotation data. Click here for file

Additional file 12: Figure S4 Expression data for key genes from tanshinone biosynthesis. **(A)** Expression profiles (RNA-seq data) of selected genes in the MVA pathway, MEP pathway, diterpenoid biosynthesis pathway, and the SmERF13 transcription factor putatively involved in regulating tanshinone production. **(B)** qRT-PCR analysis of selected genes from panel A. Error bars represent standard error relative to the mean (SEM). Click here for file

Additional file 13: Table S9 Summary of annotated cytochrome P450 genes. Click here for file

Additional file 14: Table S10 List of up- and down-regulated differentially expressed transcription factors at the various analyzed time points post induction. Click here for file

Additional file 15: Figure S5 Expression patterns for differentially expressed transcription factors (TFs) after elicitation. **(A)** Overlap of up- and down-regulated genes at each time point post induction. **(B-D)** Results from clustering the TFs according to their expression pattern, consistently down-regulated TFs **(B)**, consistently up-regulated TFs **(C)**, and TFs with inconsistent and/or minor changes **(D)**. Click here for file

Additional file 16: Table S11 Primers used for qRT-PCR analysis of selected tanshinone biosynthesis related genes. Click here for file
