# Supplementary figures and images for: Combining metabolomics and transcriptomics to characterize tanshinone biosynthesis in Salvia miltiorrhiza
Source: BMC Genomics. 2014 Jan 28;15:73. doi: 10.1186/1471-2164-15-73 (PMC3913955; doi:10.1186/1471-2164-15-73)

# Figure S1

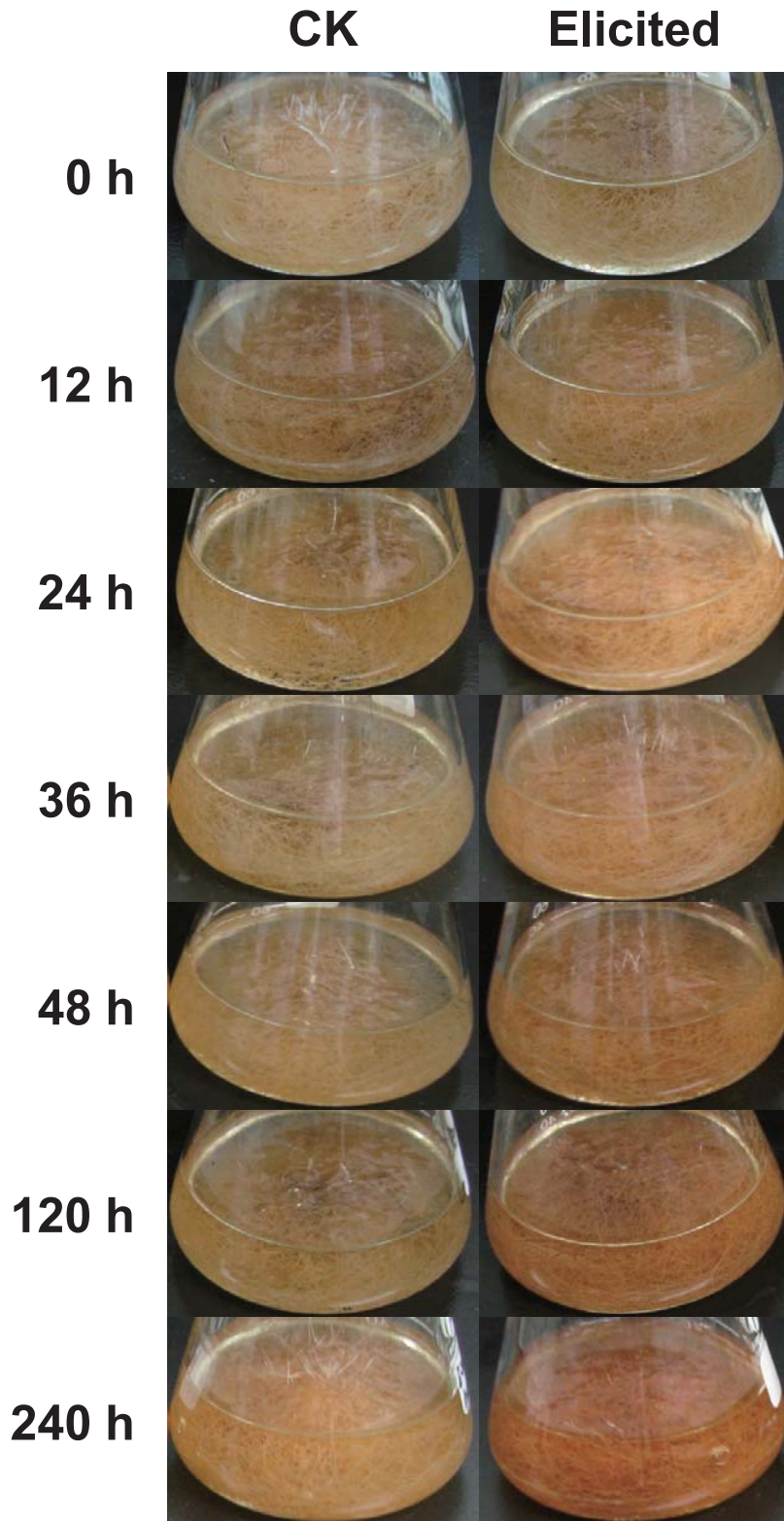

Supplement: Additional file 1: Figure S1 — Induced accumulation of tanshinones in elicited S. miltiorrhiza hairy root cultures. The observed red color is due to the accumulation of tanshinones. Thus, the notably deeper red coloration after induction relative to the control group indicates a significant increase in tanshinone content (which has been verified by LC/MS analysis – e.g. see Figures 1 and S2). [file 1471-2164-15-73-S1.pdf]

Figure S2

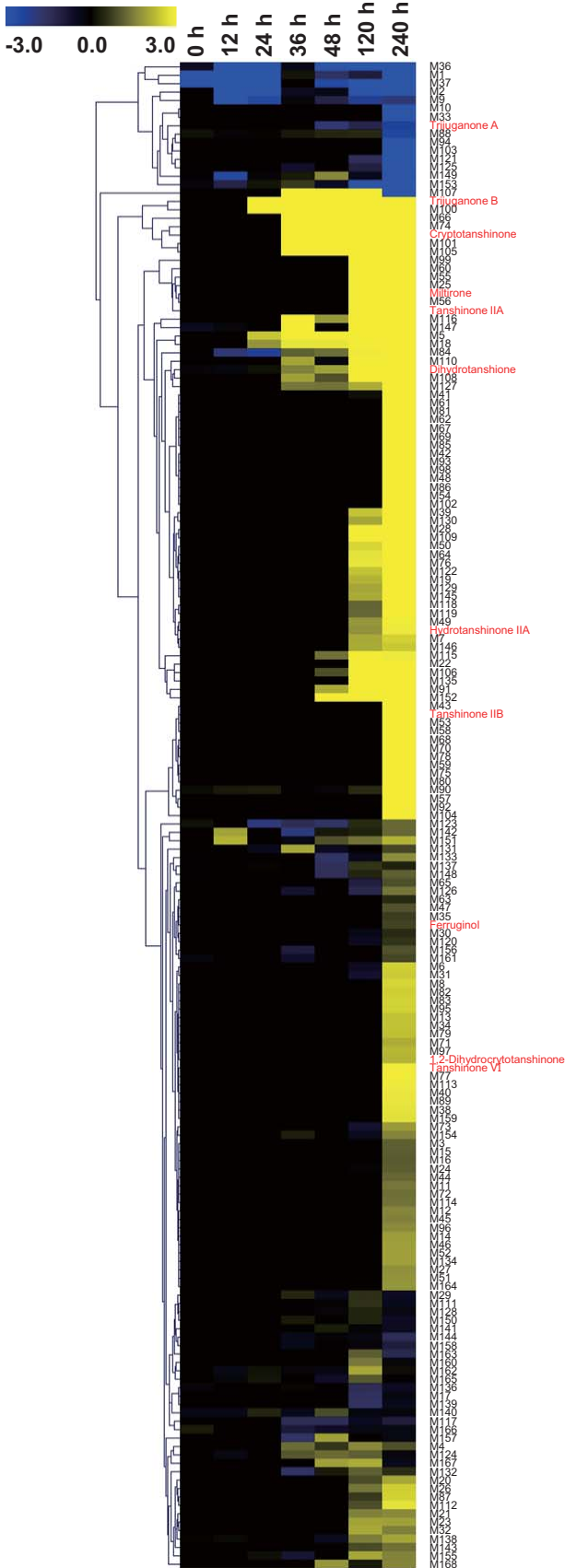

Supplement: Additional file 2: Figure S2 — Metabolomic analysis of elicited S. miltiorrhiza hairy roots by ultra-performance liquid chromatography coupled with diode array detection and quadrupole time-of-flight mass spectrometry (UPLC-DAD-QTOF-MS). A final set of 179 peaks was obtained from the metabolite profile after peak filtering analysis. The depicted heat map represents hierarchical clustering of the log-2 transformed levels for all 179 compounds. Included among these are many tanshinones related compounds (shown in red), namely miltirone, cryptotanshinone, dihydrotanshinone, trijuganone A, trijuganone B, tanshinone IIA, tanshinone IIB, tanshinone VI, ferruginol, hydrotanshinone IIA, 1,2-dihydrocrytotanshinone, etc. [file 1471-2164-15-73-S2.pdf]

Fig. S3

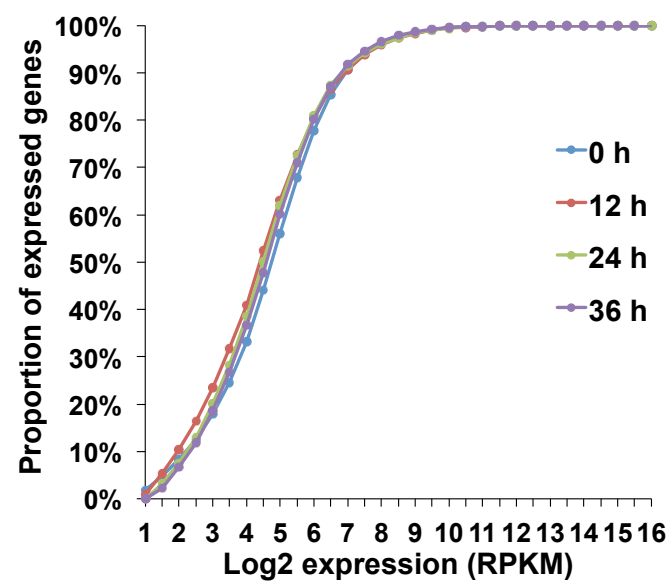

Supplement: Additional file 9: Figure S3 — Plot of gene expression abundance. The X-axis represents log-2 transformed RPKM values and the Y-axis represents the accumulating proportion of genes observed with at least that level of expression. [file 1471-2164-15-73-S9.pdf]

Figure S3

A

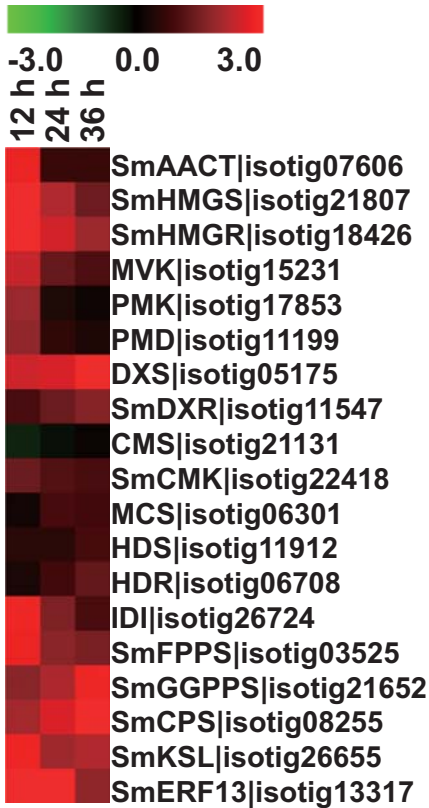

B

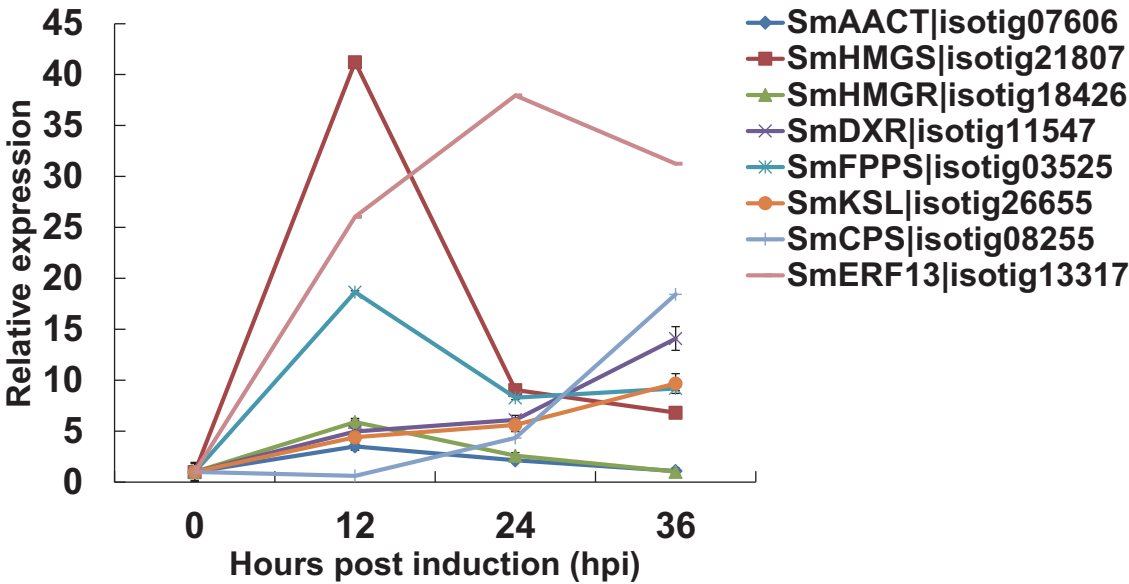

Supplement: Additional file 12: Figure S4 — Expression data for key genes from tanshinone biosynthesis. (A) Expression profiles (RNA-seq data) of selected genes in the MVA pathway, MEP pathway, diterpenoid biosynthesis pathway, and the SmERF13 transcription factor putatively involved in regulating tanshinone production. (B) qRT-PCR analysis of selected genes from panel A. Error bars represent standard error relative to the mean (SEM). [file 1471-2164-15-73-S12.pdf]

Fig. S4

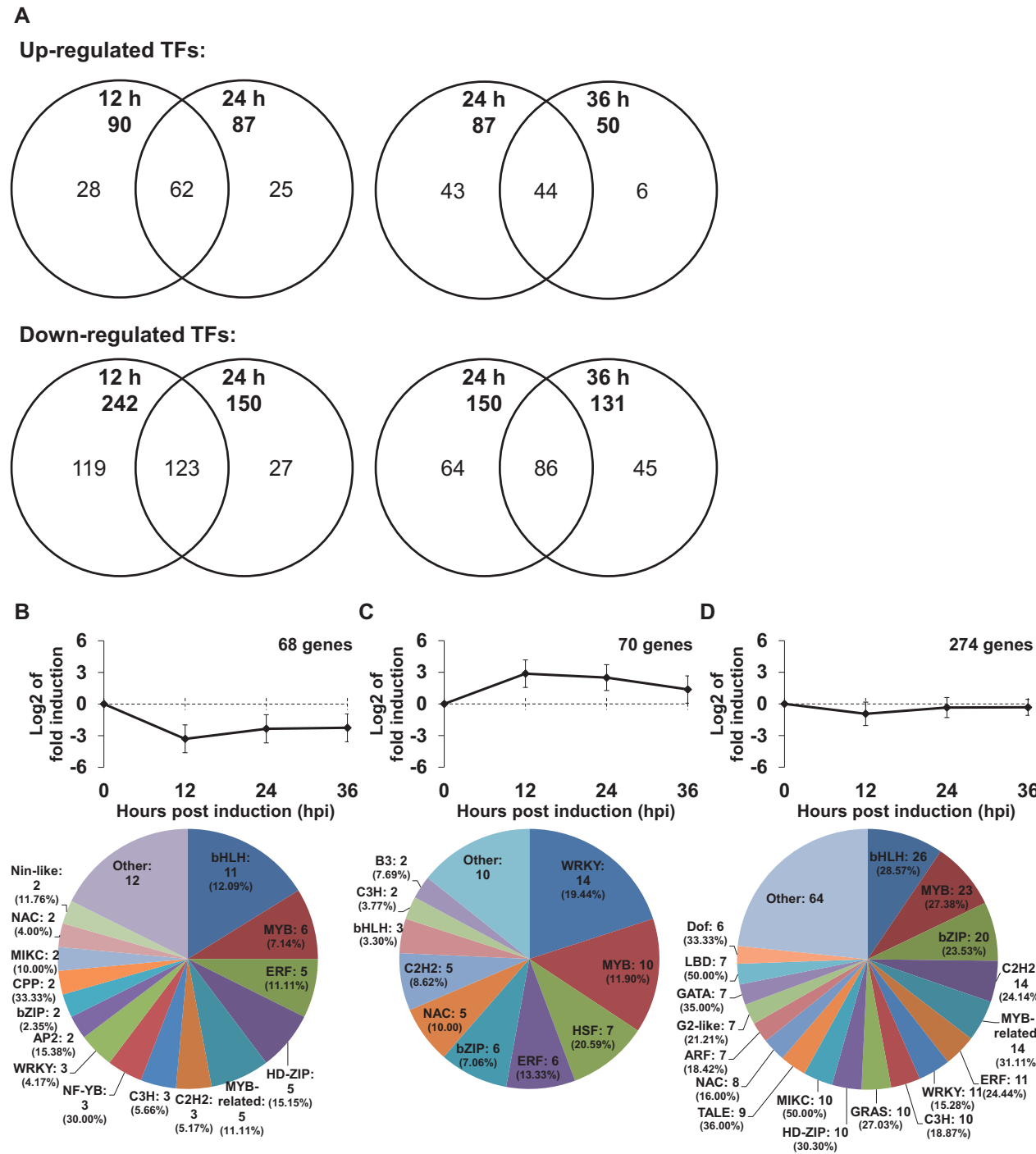

Supplement: Additional file 15: Figure S5 — Expression patterns for differentially expressed transcription factors (TFs) after elicitation. (A) Overlap of up- and down-regulated genes at each time point post induction. (B-D) Results from clustering the TFs according to their expression pattern, consistently down-regulated TFs (B), consistently up-regulated TFs (C), and TFs with inconsistent and/or minor changes (D). [file 1471-2164-15-73-S15.pdf]
