# Supplementary material for: Combining metabolomics and transcriptomics to characterize tanshinone biosynthesis in Salvia miltiorrhiza
Source: BMC Genomics. 2014 Jan 28;15:73. doi: 10.1186/1471-2164-15-73 (PMC3913955; doi:10.1186/1471-2164-15-73)
Supplement: Additional file 6: Table S4 — Summary of sequence reads with matches to the reference S. miltiorrhiza transcriptome. [file 1471-2164-15-73-S6.pdf]

Table S4: Summary of sequence reads with matches on *S. miltiorrhiza* isotigs.

Sample	Mapped reads	Total reads
0 h	5,013,001 (72.84%)	6,882,388
12 h	4,621,801 (73.36%)	6,300,372
24 h	4,321,814 (75.40%)	5,731,519
36 h	4,140,869 (72.77%)	5,690,024
