# Supplementary material for: Combining metabolomics and transcriptomics to characterize tanshinone biosynthesis in Salvia miltiorrhiza
Source: BMC Genomics. 2014 Jan 28;15:73. doi: 10.1186/1471-2164-15-73 (PMC3913955; doi:10.1186/1471-2164-15-73)
Supplement: Additional file 7: Table S5 — Summary of S. miltiorrhiza isotigs with matched sequence reads. [file 1471-2164-15-73-S7.pdf]

Table S5: Summary of *S. miltiorrhiza* isotigs with matched sequence reads.

**Sample**	**Total isotigs**	**Mapped isotigs**
0 h	25,793	20,553 (79.68%)
12 h		20,195 (78.30%)
24 h		20,305 (78.72%)
36 h		20,367 (78.96%)
Total mapped cDNAs		22,464 (87.09%)
