# Supplementary material for: Combining metabolomics and transcriptomics to characterize tanshinone biosynthesis in Salvia miltiorrhiza
Source: BMC Genomics. 2014 Jan 28;15:73. doi: 10.1186/1471-2164-15-73 (PMC3913955; doi:10.1186/1471-2164-15-73)
Supplement: Additional file 8: Table S6 — Summary of genes detected in each sample (i.e., time points post induction). [file 1471-2164-15-73-S8.pdf]

Table S6: Summary of detected genes at each induction time point.

Sample	No. of total genes	Expressed genes	Unexpressed genes
0 h	20,972	14,827 (70.70%)	6,145
12 h		14,272 (68.05%)	6,700
24 h		14,400 (68.66%)	6,572
36 h		14,300 (68.19%)	6,672
