# Supplementary material for: Combining metabolomics and transcriptomics to characterize tanshinone biosynthesis in Salvia miltiorrhiza
Source: BMC Genomics. 2014 Jan 28;15:73. doi: 10.1186/1471-2164-15-73 (PMC3913955; doi:10.1186/1471-2164-15-73)
Supplement: Additional file 10: Table S7 — List of up- and down-regulated DE genes at the various time points post induction. [file 1471-2164-15-73-S10.pdf]

Table S7: List of up- and down-regulated DE genes at different time points post-induction.

Sample	Up-regulated genes	Down-regulated genes	Total changed genes
12 h	1,537	3,619	5,156
24 h	1,240	2,418	3,658
36 h	904	1,645	2,549
