# Supplementary material for: Combining metabolomics and transcriptomics to characterize tanshinone biosynthesis in Salvia miltiorrhiza
Source: BMC Genomics. 2014 Jan 28;15:73. doi: 10.1186/1471-2164-15-73 (PMC3913955; doi:10.1186/1471-2164-15-73)
Supplement: Additional file 13: Table S9 — Summary of annotated cytochrome P450 genes. [file 1471-2164-15-73-S13.pdf]

**Table S9: Summary of annotated cytochrome P450 genes.**

| Clan | Family | Subfamily | Member |
|---|---|---|---|
| CYP51 clan | CYP51 | CYP51G | 2 |
| CYP71 clan | CYP701 | CYP701A | 2 |
| | CYP706 | CYP706A | 3 |
| | CYP71 | CYP71A | 2 |
| | | CYP71B | 17 |
| | CYP73 | CYP73A | 1 |
| | CYP75 | CYP75B | 3 |
| | CYP76 | CYP76C | 9 |
| | | CYP76G | 2 |
| | CYP78 | CYP78A | 2 |
| | CYP79 | CYP79B | 1 |
| | CYP81 | CYP81D | 2 |
| | | CYP81F | 1 |
| | | CYP81H | 1 |
| | CYP82 | CYP82C | 2 |
| | | CYP82F | 1 |
| | | CYP82G | 1 |
| | CYP83 | CYP83B | 4 |
| | CYP84 | CYP84A | 4 |
| | CYP89 | CYP89A | 1 |
| | CYP98 | CYP98A | 5 |
| CYP710 clan | CYP710 | CYP710A | 1 |
| CYP711 clan | CYP711 | CYP711A | 3 |
| CYP72 clan | CYP714 | CYP714A | 3 |
| | CYP715 | CYP715A | 1 |
| | CYP72 | CYP72A | 17 |
| | | CYP72C | 1 |
| | CYP721 | CYP721A | 1 |
| | CYP735 | CYP735A | 2 |
| CYP85 clan | CYP707 | CYP707A | 1 |
| | CYP716 | CYP716A | 9 |
| | CYP88 | CYP88A | 3 |
| | CYP90 | CYP90A | 1 |
| | | CYP90C | 1 |
| | | CYP90D | 1 |
| CYP86 clan | CYP704 | CYP704A | 3 |
| | CYP86 | CYP86A | 2 |
| | CYP94 | CYP94B | 1 |
| | | CYP94C | 1 |
| | | CYP94D | 2 |

| | | | |
|---|---|---|---|
| | CYP96 | CYP96A | 1 |
| CYP97 clan | CYP97 | CYP97B | 3 |
| | | CYP97C | 1 |
