# Supplementary material for: Combining metabolomics and transcriptomics to characterize tanshinone biosynthesis in Salvia miltiorrhiza
Source: BMC Genomics. 2014 Jan 28;15:73. doi: 10.1186/1471-2164-15-73 (PMC3913955; doi:10.1186/1471-2164-15-73)
Supplement: Additional file 14: Table S10 — List of up- and down-regulated differentially expressed transcription factors at the various analyzed time points post induction. [file 1471-2164-15-73-S14.pdf]

Table S10: List of up- and down-regulated differentially expressed transcription factors at different time points after induction.

Sample	Up-regulated	Down-regulated	Subtotal
12 h	90	242	332
24 h	87	150	237
36 h	50	131	181
