# Supplementary material for: Combining metabolomics and transcriptomics to characterize tanshinone biosynthesis in Salvia miltiorrhiza
Source: BMC Genomics. 2014 Jan 28;15:73. doi: 10.1186/1471-2164-15-73 (PMC3913955; doi:10.1186/1471-2164-15-73)
Supplement: Additional file 16: Table S11 — Primers used for qRT-PCR analysis of selected tanshinone biosynthesis related genes. [file 1471-2164-15-73-S16.pdf]

Table S11: Primers used for qRT-PCR analysis of selected tanshinone biosynthesis related genes.

Gene Name	Primer sequence (5′-3′)
SmAACT	SmAACT-1: ATGCTGAAGGACGGACTCTGGGATG<br>SmAACT-2: TTGTCAACAATGGTGGATGG
SmHMGS	SmHMGS-1: GATGCCGACTACTTTGTATTTC<br>SmHMGS-2: CTCGACTTCAACTTCTCTGAA
SmHMGR	SmHMGR-1: GCAACATCGTCTCCGCCGTCTACA<br>SmHMGR-2: GATGGTGGCCAGCAGCCTGGAGTT
SmDXR	SmDXR-1: GAGAATCTACTGCTCCGAGA<br>SmDXR-2: CTGGTCGTAGTGGATGATCT
SmFPPS	SmFPPS-1: TTTTACCTCCCAGTTGCTTGTG<br>SmFPPS-2: TTTACAACCAGCCAAGAACATT
SmKSL	SmKSL-1: CTTCCCAAGACAATGCAAAGAT<br>SmKSL-2: ATTTCCCTCTCACATTATTAGC
SmCPS	SmCPS-1: GATCGGAAGACGCTGTA<br>SmCPS-2: TCGCCAAGAAATAGGAAA
SmERF13	SmERF13-1: GAAATTCGCGGCGGAGATACGG<br>SmERF13-2: TCGCTTACTCACCCTCGTCGGCT
Actin	Actin-1: AGGAACCACCGATCCAGACA<br>Actin-2: GGTGCCCTGAGGTCCTGTT
